# Evaluation of microbial contamination in multi-dose fluorescein eyedrops in a reference eye center

**DOI:** 10.5935/0004-2749.20210070

**Published:** 2021

**Authors:** Camila de Moraes Costa, Kayo Pinheiro Espósito, Victor Medeiros Brandão Florêncio, Paulo Romero Leite Aquino, Elza Ferreira Firmo, Iggor Macêdo do Amaral Costa, Camilla Rocha, Camila Galvão de Andrade, Jorge Belém Oliveira-Júnior, Camila V. Ventura

**Affiliations:** 1 Department of Ophthalmology, Fundação Altino Ventura, Recife, PE, Brazil; 2 Laboratory of Molecular Biology and Bacterial Genetics, Department of Tropical Medicine, Universidade Federal de Pernambuco, Recife, PE, Brazil; 3 Laboratory of Molecular and Celular Biology, Department of Parasitology, Instituto Aggeu Magalhães, Recife, PE, Brazil; 4 JEA Laboratório de Análises Clínicas, Timbaúba, PE, Brazil; 5 Department of Ophthalmology, Hospital de Olhos de Pernambuco, Recife, PE, Brazil

**Keywords:** Fluorescein, Ophthalmic solution, Drug contamination, Eye infection, bacterial/microbiology, Bacteria/isolation & purification, Fluoresceína, Soluções oftálmicas, Contaminação de medicamentos, Infecções oculares bacterianas/microbiologia.

## Abstract

**Purpose:**

To analyze the presence of microorganisms in fluorescein eyedrops used in a reference eye center in Recife-PE.

**Methods:**

This real-life and masked study evaluated fluorescein eyedrops used at the Altino Ventura Foundation in May 2019. Cultures were performed according to exposure times; I) three eyedrop bottles were analyzed after one day of use, II) three eyedrop bottles after 4 d of use, III) three eyedrop bottles after 8 d of use, and IV) three unopened bottles used as control. Samples were collected from the bottle’s tip, instilled drop, and residual fluid. After incubation, all colonies were analyzed and identified through biochemical tests.

**Results:**

The contamination rate of the fluorescein eyedrop bottles in this study was 55.5% (5/9 vials). There was no contamination in the control group. The highest contamination was seen in one day exposed eyedrops, in 100% of the bottles. The bottle’s tip had a higher rate of contamination compared to the drop and residual fluid. Gram-positive bacteria were isolated in 7/27 (25.9%) samples. Growth of fungi or gram-negative bacteria was not observed.

**Conclusion:**

The identification of gram-positive bacteria predominantly on the tip of the fluorescein eyedrop bottles suggests inadequate handling as the main cause of contamination.

## Abstract (pt)

**Objetivo:**

Analisar a presença de microrganismos nos colírios de fluoresceína utilizados em um centro oftalmológico de referência em Recife-PE.

**Métodos:**

Este estudo de vida real e mascarado avaliou colírios de fluoresceína utilizados na Fundação Altino Ventura em maio/2019. As culturas foram realizadas de acordo com os diferentes tempos de exposição: I - três frascos de colírio foram analisados após 1 dia de uso; II - três frascos de colírio após 4 dias de uso; III - três frascos de colírio após 8 dias de uso; IV - três garrafas fechadas foram usadas como grupo controle. As amostras foram coletadas da ponta do frasco, da gota instilada e do líquido residual interior. Após incubação, todas as colônias foram analisadas e identificadas através de testes bioquímicos.

**Resultados:**

A taxa de contaminação dos frascos de colírio de fluoresceína neste estudo foi de 55,5% (5/9 frascos). Não houve contaminação no grupo controle. A maior contaminação foi observada os colírios expostos de um dia - 100% dos frascos. A ponta da garrafa teve uma maior taxa de contaminação em comparação com as culturas de gota e de fluido residual inferior. Bactérias gram-positivas foram isoladas em 7/27 amostras (25,9%). Não houve crescimento de fungos ou bactérias Gram-negativas.

**Conclusão:**

A identificação de bactérias Gram-positivas predominantemente na ponta dos frascos de colírio de fluoresceína sugere manuseio inadequado como a principal causa de contaminação de colírios multidose.

## INTRODUCTION

In ophthalmological practice, the same eyedrop bottle is routinely used to administer drops to several patients. Although the no-touch technique is used, sometimes touch becomes inevitable, leading to eyedrop contamination, and increasing the risk of cross-infection^(1)^. Eyedrop contamination has been reported in the literature ranging from very low rates (0.25%) to considerably high rates (16.8%)^(1,2)^.

Eye infection by pathogens transmitted through reused eyedrop bottles can lead to keratitis and corneal ulcers, with risk of transmission of opportunistic and pathogenic microorganisms such as *Pseudomonas aeruginosa* and *Serratia marcescens*, which may interfere with the pH of the drug and, consequently, its metabolism and efficacy^(3,4)^. Most studies found commensal microorganisms (predominantly gram-positive bacteria) with low-infectivity from ocular and skin microbiota^(1-3)^. Because fluorescein solutions are among the most used ophthalmic preparations, their contamination with pathogenic bacteria has been studied extensively^(5)^.

Gram-negative bacterial contamination of eye drops represents a potentially serious risk for eye infections, especially in ocular surface disease, after intraocular surgery with wound leakage, or in corneal epithelial damage, such as extensive use of contact lens, eye trauma, or use of topical steroids^(3,6)^. The transmission of bacterial eye infection, such as keratitis and endophthalmitis, by a contaminated dropper has been reported^(5)^. Moreover, the transmission of lacrimal film viruses, ranging from adenoviruses (common in ophthalmic practice) to HSV-1, varicella zoster virus, cytomegalovirus (CMV), Epstein- Barr virus, hepatitis B and C, and HIV from infected patients has been reported^(7)^.

Preservatives are used in eye drops to reduce microbial proliferation. However, they can cause irritation, allergy, and ocular surface disorders^(6)^. The antimicrobial efficacy, concentration, or duration of action of preservatives has been questioned due to the growth of strains after repeated use^(2,3)^. Thus, a balance between ocular toxicity and antimicrobial efficacy for preservatives is required. Moreover, the preference and safety of single-dose containers for administering eye drops to reduce infection risk and need for preservatives should be discussed^(2)^.

The financial cost and environmental impact for reducing infection risk to zero by using individual disposable droppers are significant and justify the practice of reuse of eyedrop bottles. Measures should be taken to minimize contamination and transmission, such as restricting the use time at home and in the hospital, recording the date of opening of the container, and spreading awareness at work by educating the handlers, including the employees, doctors, patients, or caregivers^(3)^.

We have evaluated the presence of microorganisms in fluorescein eyedrops in an outpatient ophthalmology clinic due to the high contamination rates in the hospital environment.

## METHODS

This real-life and masked study analyzed 1% fluorescein eyedrops samples used routinely by the outpatients of Altino Ventura Foundation, Recife/PE, in May 2019. Ophthalmologists were unaware of the study and the way the bottles were handled was not supervised by the researchers.

The collection was performed at different exposure times, which are as follows: I) three vials exposed during one day of use 24 hours (h); II) three vials exposed during 4 days (d) of use (96 h); III) three vials exposed during 8 d of use (192 h); IV) three closed eyedrops bottles, which were used as controls. All eyedrop bottles were from the same lot and manufacturer and were opened and released for use in the same week and outpatient sector (except the control group). Information related to the study was not disclosed to the users and health care professionals at the site.

After the exposure period, the vials were transported for processing. Samples were collected from the residual fluid of the vial and the container tip through sterile swabs and from the drop, which was deposited directly into the culture medium.

Initially, all samples were cultured in the brain heart infusion (BHI) broth and incubated at 36ºC ± 1. After 18-24 h, the cultures were seeded on BHI agar, 5% sheep blood agar, and MacConkey agar. In addition, the BHI broth was incubated for an additional 48h at 36ºC ± 1, placed on Sabouraud dextrose agar, and incubated for at least 7 d at 36ºC ± 1. In cases where there was no growth in this period, the plates were incubated for a further 48h to confirm the result. Plates without apparent colonies were incubated again for 30 d to ensure sufficient growth time was given to some fungal species and to confirm the results. Thereafter, the isolates were submitted for phenotypic identification through biochemical tests.

Regarding the methodological analysis, categorical variables were expressed as their absolute and relative frequencies.

## RESULTS

Control eyedrops showed no growth. However, bacterial growth occurred in 7/27 samples from the eyedrops exposed to use, representing a contamination rate of 25.9% (Table 1).

**Table 1:** Culture of microbial species isolated from contaminated eyedrops in an outpatient eye hospital

Groups	Sample Site		
	Bottle Tip	Drop	Residual content
Control			
Bottle A	-	-	-
Bottle B	-	-	-
Bottle C	-	-	-
1 -day exposure			
Bottle A	**S.** *epidermidis*	-	-
Bottle B	*S. aureus*	-	-
	CoNS		
Bottle C	**S.** *epidermidis*	CoNS	CoNS
4-day exposure			
Bottle A	-	-	-
Bottle B	**5.** *saprophyticus*	-	-
Bottle C	-	-	-
8-day exposure			
Bottle A	-	-	-
Bottle B	-	-	-
Bottle C	*S. aureus*	-	-
	*S. saprophyticus*		

(-) = No microbial growth; CoNS= Coagulase-negative staphylococci.

The evaluation of the vial’s contamination site revealed bacterial growth in the tip of 5/9 vials (55.5%) and in one vial (11.1%) both the drop and the residual vial showed bacterial growth. The identified microorganisms in these vials were all gram-positive bacteria. No growth of gram-negative bacteria or fungi was observed.

**Figure 1:**
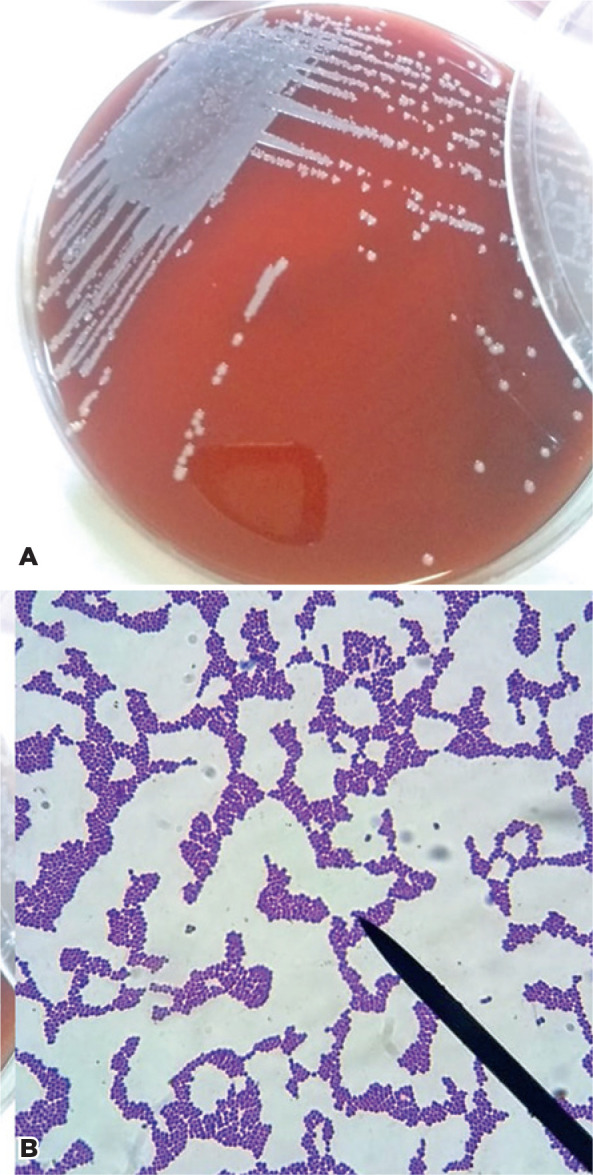
*Staphylococcus* sp. colonies on 5% sheep blood agar (A) and co lonies of gram-positive bacteria, identified by Gram’s method (B) obtain ed from fluorescein eyedrops in an outpatient eye hospital.

In eyedrops exposed for one day, five contaminated samples (55.5%) were observed. Gram-positive bacteria grew at the tip of three vials. At the tip of bottle A, growth of *Staphylococcus epidermidis* and at the tip of bottle B, growth of two types of gram-positive microorganisms, *S. aureus* and Coagulase-negative *staphylococci* (CoNS), was observed. In a bottle exposed for one day (vial C) growth of *S. epidermidis* at the tip and CoNS in both the drop and at the bottom of the bottle (Figure 1) was observed.

The eyedrops exposed for 4 d presented contamination of 11.1% of the samples. Only one bottle showed *S. saprophyticus* growth at its tip, but without contamination of the drop or residual content. No microbial growth was identified in the other two bottles (66.6% of samples).

The group exposed for 8 d presented a similar contamination rate as the group exposed for 4 d, representing 11.1% of the samples. The growth of two gram-positive microorganisms, *S. aureus* and *S. saprophyticus*, was observed at the tip of one bottle and no contamination of the drop or vial residual content was detected. No microbial growth was identified in the other two bottles (66.6% of the sample).

## DISCUSSION

The risk of microorganism transmission by contaminated eyedrops has been described in the literature^(3)^. The finding of bacterial contamination suggests the presence of other pathogens not evaluated in this study, such as fungi and viruses^(2,3,7)^. To study the presence of microorganisms, it is recommended to collect samples from different locations of the container and to identify the pathogens, as when detected in significant numbers in different locations, they indicate infection risk^(2)^. In the present study, three sites for each eye drop bottle were analyzed (bottle tip, drop, and residual content), similar to other studies^(2,3)^.

A study conducted in the United Kingdom estimated that the risk of cross-contamination with eyedrops is approximately 1:400 with single reuse, reaching 1:80 if used more than six times^(1)^. Although the use of single use eyedrops is recommended, it has high financial and environmental cost^(3)^.

To our knowledge, no study has compared the contamination rate of fluorescein eyedrops used in ophthalmology and correlated its relationship with the number of days in use in the same ophthalmologic center. In this study, contamination rate was identified in 55.5% of bottles, which was higher than that found in other studies. Teuchner et al. found a 17.1% contamination rate in mydriatic and anesthetic eyedrops after a 4-week period of use^(2)^. Another author found a contamination rate of 38% in non-antibiotic eyedrops after 7 d of use in outpatients^(6)^. However, the only study that evaluated fluorescein eyedrops found a 100% contamination rate. However, the bottles were collected from various eye centers in Ghana and the period of use was not specified^(8)^.

As found in other studies, the main site of contamination in the current study was the tip of the bottle^(3,9)^. However, it is important to highlight that none of these studies specifically analyzed fluorescein eyedrops or exposure time similar to this study. The only study that was similarly conducted in an outpatient eye clinic setting did not find a statistical difference between the contamination rates at the tip and other sites (drop and residual content)^(2)^.

The contamination of tips can be explained by the inappropriate handling of the bottles, caused either by direct contact with the patient’s eyelids or by leaving the bottle uncapped and exposed during the day. In fact, only one eyedrop bottle (11.1%) presented contamination of the residual content of the bottle and the drop.

Prolonged exposure of the eyedrop is expected to increase the contamination rate. However, a higher contamination rate of eyedrops exposed for 1 d compared to those exposed for 4 and 8 d was observed. Immediately after the experiment, some physicians reported the use of alcohol to clean the eyedrop tip before its use when they noticed it was opened from the day before. The use of 70% alcohol is effective as an antimicrobial agent in the hospital^(10)^. This procedure is not an institutional policy but a common habit of some professionals in the eye center. Furthermore, it is noteworthy that the researchers did not interfere in the use of eyedrop bottles by the professionals. Also, the high rate of eyedrop contamination used on the first day may be related to a less cautious use of newly opened eyedrops by the professionals, resulting in touching of the eyelids during eye drop administration.

This study did not consider the number of times the bottle was used for administering eyedrops, the number of patients that the bottle was used on, the period of time that the bottles were uncapped, and how each physician handled the eyedrop bottle. Thus, further studies are necessary to clarify the relationship between these variables and eyedrop contamination rate for standardizing safety procedures during eyedrop use in an outpatient setting.

Similar to other studies, the microbiological contamination profile in this study revealed a prevalence of skin and conjunctiva flora and microorganisms present in the environment^(1-3,7)^. The gram-positive bacteria identified included *S. epidermidis, S. aureus, Coagulase-negative staphylococci* (CoNS), and *S. saprophyticus*. Although the first three microorganisms are part of the skin and conjunctival microbiota, they have a pathogenic potential and are responsible for most eye infections, including blepharitis, conjunctivitis, keratitis, corneal ulcer, endophthalmitis, and orbital cellulitis^(11)^. *S. saprophyticus*, on the other hand, is usually an opportunistic germ related to genitourinary tract infections and no eyedrop contamination by this microorganism has been reported in similar studies^(12)^. In addition, no fungal growth was identified in our samples, which corroborates with previous studies by Nentwich et al. and Schein et al.^(3,4)^.

The limitations of this study included sample size and non-observation of eyedrop manipulation by the researchers for better analysis of factors, including the use of 70% alcohol, which may influence the contamination of the bottle tips. Nevertheless, the identification of gram-positive bacteria predominantly at the tip of the bottles suggests inappropriate use as the main source of multi-dose eyedrop contamination. Thus, further studies should be conducted to evaluate the effectiveness of educational measures and the impact of antiseptic use for the effective control of eyedrop contamination.
